# Deciphering the Anti-LUAD Mechanism of 4′-Demethyl-epipodophyllotoxin (4′-DMEP) via Machine Learning-Driven Target Identification and In Vitro Validation

**DOI:** 10.1021/acsomega.5c12467

**Published:** 2026-04-03

**Authors:** Jinghui Yao, Chenhao Wang, Zhichao Wang, Shi Xiang, Wu Sun, Hui Chen, Chao Yang

**Affiliations:** † Oncology Research Center, Jiangxi Provincial Key Laboratory of Traditional Chinese Medicine Diagnosis and Rehabilitation of Malignant Tumors, 74582 Jiangxi University of Traditional Chinese Medicine, Nanchang 330004, P. R. China; ‡ Jiangxi Engineering Research Center for Translational Cancer Technology, Jiangxi University of Traditional Chinese Medicine, Nanchang 330004, P. R. China

## Abstract

4′-Demethyl-epipodophyllotoxin (4′-DMEP) is a precursor of etoposide and has attracted much attention due to its significant antitumor activity. In order to elucidate the molecular mechanism of its inhibition of lung adenocarcinoma (LUAD), this study comprehensively used network pharmacology, machine learning, and molecular simulation technology, and verified it through cell experiments. A total of 171 drug targets and 11,439 disease targets were collected from the public database, and 131 intersection targets were obtained using the Venny tool. Then the 131 targets were visualized by PPI network. Subsequently, 38 significantly differentially expressed targets were screened and identified in the GEPIA database, from which 18 survival-related genes were further screened. GO functions related to mitotic cell cycle regulation and ERK1/ERK2 signaling, as well as KEGG pathways including gap junction and infection-related pathways, were also enriched. Four machine learning models screened five characteristic genes (SLC2A1, TOP2A, MIF, TLR4, and PLA2G1B). Molecular docking confirmed high-affinity interactions (binding energies ≤ −6.1 kcal/mol), a finding that was further validated by molecular dynamics simulation. In vitro experiments revealed that 4′-DMEP inhibited A549 cell proliferation by inducing cell cycle arrest and apoptosis, with significant changes in the expression of SLC2A1, TOP2A, and MIF. These findings not only clarify its molecular mechanism but also provide new ideas for the precise treatment of lung adenocarcinoma.

## Graphical abstract

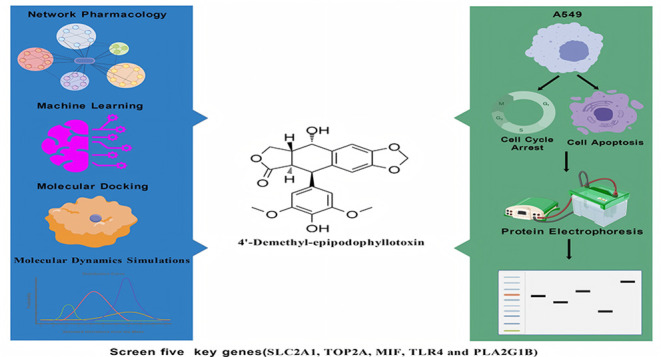

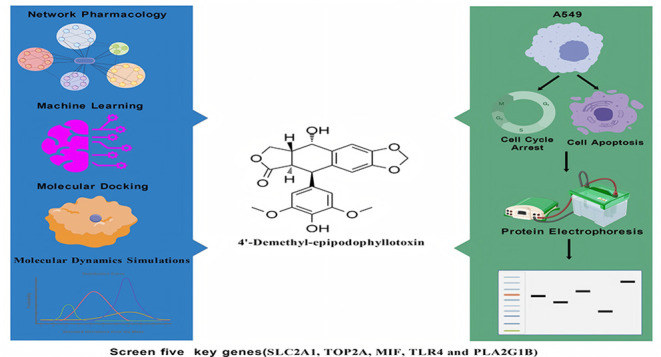

## 1 Introduction

Lung adenocarcinoma (LUAD) is a major subtype of non-small cell lung cancer (NSCLC). Despite advances in targeted therapy, the 5-year survival rate for patients with advanced disease remains low, placing a heavy burden on global health.^1,2^ The development of resistance and tumor heterogeneity remains a significant challenge in the treatment of LUAD, highlighting the urgent need for novel therapeutic agents.^3,4^ In the face of the above clinical challenges, natural product derivatives have become the key direction for exploring anti-LUAD drug lead compounds in the field of scientific research due to their unique advantages of structural diversity and excellent biological activity, which provides a key idea for breaking through the existing treatment bottleneck.^5^ Among them, 4′-demethyl-epipodophyllotoxin (4′-DMEP) has attracted attention due to its unique structural characteristics and demonstrated antitumor potential.^6^

4′-DMEP (C_21_H_20_O_8_, MW: 400.38 Da) is a naturally occurring lignan compound isolated from plants of the genera Podophyllum and its close relatives Sinopodophyllum and Dysosma, which belong to the family Berberidaceae.^7^ These plants have a long history of use in traditional medicines, such as Tibetan and Chinese folk medicine, for treating skin diseases, inflammatory conditions, and even tumors. Structurally, 4′-DMEP is characterized by a four-ring core (containing two benzene rings, a lactone ring, and a dioxolane) and a 4′-position demethyl group—a key structural modification. 4′-DMEP exerts microtubule-targeting activity by enhancing the binding to β-tubulin through demethylation.^8^ Its tetracyclic core disrupts microtubule dynamics, thereby inhibiting mitosis and tumor proliferation.^9^ As a precursor to etoposide/teniposide, 4′-DMEP has broad anticancer potential.^10^ However, its molecular mechanisms in LUAD are still poorly understood. A plethora of studies have hitherto been conducted on the subject, the majority of which have focused on the general anticancer effects of the subject in question. However, there has been a paucity of in-depth exploration of its specific action mechanisms in LUAD, particularly comprehensive validation that bridges target prediction, molecular dynamics simulations, and functional assays. This knowledge gap has hindered its translation into precision oncology for the treatment of LUAD.

Traditional target discovery has the characteristics of a long time span, high cost, and high failure rate. In recent years, machine learning (ML) has emerged as a powerful tool to address such knowledge gaps in drug discovery and mechanism elucidation.^11^ ML refers to a subset of artificial intelligence that enables computer systems to learn patterns from large data sets, make predictions, and optimize models without explicit programming. ML has revolutionized the process of therapeutic target recognition by using a large number of biomedical data sets to decipher complex patterns.^12^ The central premise of ML-driven target recognition is to reframe the discovery process as a predictive modeling task. Specifically, given a specific disease state (e.g., LUAD, etc.), the ML algorithm is used to predict which candidate genes among thousands of genes or proteins are most likely to be effective and safe therapeutic targets.

In this paper, we designed a multidisciplinary research framework. Network pharmacology and ML were employed to identify LUAD-associated proteins that may interact with 4′-DMEP, thereby providing a theoretical basis for subsequent experiments. Molecular docking and dynamics simulations (MD&D) were then conducted to evaluate the binding stability of 4′-DMEP with the identified proteins, helping to clarify the molecular-level interaction mechanisms.^13^ In vitro validation experiments were performed in A549 cells to directly observe the effects of 4′-DMEP on LUAD cells.

## 2 Materials and Methods

### 2.1 Target Prediction and Screening

The SMILES notion of 4′-DMEP COC1=CC­(=CC­(=C1O)­OC)­[C@H]­2­[C@@H]­3­[C@H]­(COC3=O)­C@ @H­(C4=CC5=C­(C=C24)­OCO5)O was retrieved from PubChem (https://pubchem.ncbi.nlm.nih.gov) and subsequently submitted to TargetNet (http://target-net.scbdd.com) to identify potential target genes through their corresponding UniProt IDs. These UniProt IDs were then cross-referenced in the UniProt database (https://www.uniprot.org) to obtain official gene nomenclature. Parallelly, target prediction was performed using the SuperPred database (https://prediction.charite.de) with the same SMILES input. For the compound 4′-DMEP, its InChI code, acquired from PubChem, was used as a query in the BATMAN database (http://bionet.ncpsb.org/batman-tcm) to screen for potential therapeutic targets.

LUAD-associated targets were systematically collected through multiple databases, such as GeneCards (https://www.genecards.org), OMIM (https://www.omim.org), and Therapeutic Target (TTD, http://db.idrblab.net/ttd). Finally, we used Venny 2.1.0 (https://bioinfogp.cnb.csic.es/tools/venny/index.html) to identify overlapping targets between 4′-DMEP and LUAD.^14^

### 2.2 Screening of Core Targets

The GEPIA database (http://gepia.cancer-pku.cn) was used for the screening of all intersection targets to identify genes exhibiting significant differences. We used the criteria of | log2 fold change | > 1 and adjusted *p* < 0.05 to query 131 intersection targets for LUAD samples in the GEPIA database. Genes that met these thresholds were identified as significantly differentially expressed, resulting in 38 genes. From these 38 genes, we further filtered for those associated with survival by applying a survival curve threshold of *p* < 0.05 (Log-Rank p, p (HR)). The genes satisfying this condition were designated as the final core genes, totaling 18. The subsequent step involved the utilization of Cytoscape 3.8.0 to construct and visualize the associated protein–protein interaction (PPI) network.^15^

### 2.3 GO Function and KEGG Enrichment Analysis

Gene Ontology (GO) enrichment analysis was performed on all intersection targets. The biological processes (BP), cellular components (CC), and molecular functions (MF) of these targets were then retrieved from the DAVID database (DAVID: Functional Annotation Result Summary). The top ten terms in each category were then filtered based on *p*-value, and an enrichment analysis was conducted on the Weishengxin platform. The generation of bar plots followed this. Additionally, KEGG pathway enrichment analysis was to be performed for these intersection targets using the Bioinformatics.com.cn platform (http://www.bioinformatics.com.cn/).

### 2.4 GEO Sample Collection and Processing

A search was conducted on the GEO database using the keyword “lung adenocarcinoma”, with the data type and organism (*Homo sapiens*) parameters restricted. This yielded three data sets, namely, GSE18842,^16,17^ GSE19188,^18,19^ and GSE31210,^20,21^ which were selected for the acquisition of gene expression and clinical data. For analysis and model construction, we integrated two data sets (GSE18842 and GSE19188). Batch effects were removed using the “ComBat” function. GSE31210 was used for the external model validation. Perl code was then used for gene symbol annotation and data correction to obtain the expression levels of core genes in each sample of the normal group and the LUAD group in network pharmacology.

### 2.5 Differential Expression, Genomic Loci Mapping, and Expression Correlation of Core Genes

Expression levels of core genes in normal and LUAD cohorts were quantified, followed by differential expression analysis using R packages limma, pheatmap, and ggpubr. Significantly differentially expressed core genes (SDECGs) were identified through boxplot visualization and hierarchical clustering heatmaps (Benjamini-Hochberg adjusted *p* < 0.05). Chromosomal loci mapping was performed via custom Perl scripts, with genomic coordinates visualized as Circos plots using the circlize R package. Pairwise expression correlations between SDECGs were computed via Pearson’s r implemented in base R cor (4.4.1) function (two-tailed, α = 0.05).^22^

### 2.6 Infiltration and Correlations of Immune Cells in LUAD

The CIBERSORT command in the R language was utilized to calculate the relative abundance of immune cells through 1,000 simulations. Single-sample gene set enrichment analysis (ssGSEA) was performed to compare the difference in immune cell content between the normal group and the LUAD group. The ssGSEA results were presented as boxplots. The SDECGs were then intersected with the ssGSEA scores, and correlation tests were performed to obtain correlation coefficients, which were then visualized.^23^

### 2.7 Development and Validation of Machine Learning Models

Four machine learning models were developed to classify samples based on the expression profiles of predefined SDECGs, including Random Forest (RF), Support Vector Machine with a radial basis function kernel (SVM), Generalized Linear Model with logistic regression (GLM), and eXtreme Gradient Boosting (XGB).^24,25,28^ All analyses were performed in R (version 4.4.1) using the caret package, with algorithm implementations provided by the random Forest, kernlab, and xgboost packages. Importantly, feature selection was conducted prior to model training. A fixed set of SDECGs was defined based on independent differential expression analysis and was consistently used for all subsequent modeling procedures. No additional feature selection was performed within cross-validation folds, thereby ensuring that model training and evaluation were not influenced by test-set information. The data set was randomly divided into a training set (70%) and an independent testing set (30%). Model training and hyperparameter optimization were performed exclusively on the training set using repeated 5-fold cross-validation (five-folds with five repeats), as implemented by the trainControl function in caret. This resampling strategy was adopted to provide robust internal performance estimation while minimizing overfitting.

For the RF model, the number of variables randomly sampled at each split (mtry) was optimized, while the number of trees was fixed at 500. For the SVM model, a radial basis function kernel was applied, and the cost parameter (C) and kernel width (sigma) were tuned over default parameter grids, with sigma initialized using the suggest function in the kernlab package. The GLM model was fitted as a logistic regression without additional hyperparameter tuning using maximum likelihood estimation. The XGB model was trained using the xgbDART algorithm, incorporating dropout regularization, with tuning of key parameters including the number of boosting rounds (nrounds), maximum tree depth (max_depth), learning rate (eta), and dropout rate (rate_drop). The final model for each algorithm was selected based on the highest area under the receiver operating characteristic curve (AUC) achieved during cross-validation on the training set. Model performance was subsequently evaluated on an independent testing set. ROC curves and AUC values were computed by using the pROC package. To further assess predictive stability and mitigate concerns of overfitting, complementary performance metrics (including residual boxplots, reverse cumulative distribution plots, and confusion matrix)-derived classification indices (accuracy, sensitivity, specificity, precision, and F1 score) were jointly considered. Permutation-based variable importance was estimated post hoc using the DALEX package for model interpretation only and did not influence model training. The top five variables ranked by importance from the optimal model were used to construct a multivariable nomogram. Calibration curves and decision curve analysis (DCA) were applied to evaluate the model calibration and clinical utility.

To further assess generalizability, the entire modeling pipeline was applied to an independent external validation cohort obtained from the GEO database (GSE31210). Predictive performance in the external data set was evaluated using ROC analysis and compared with that of the internal testing set.

### 2.8 Molecular Docking and Molecular Dynamics Simulations

The three-dimensional structure of 4′-DMEP (PubChem CID: 122797) was retrieved from the PubChem database, and the target protein structure was obtained from UniProt (https://www.uniprot.org/) (Supporting Table S1). AutoDock Vina 1.2.3 was used for molecular docking to verify the core network pharmacology hypothesis, and PyMOL 2.5.7 was used for the binding energy calculation and pharmacophore interaction visualization.

Molecular dynamics (MD) simulations were conducted by utilizing the GROMACS 2022 program, employing the GAFF force field for small molecules, the AMBER14SB force field for proteins, and the TIP3P water model. The combination of protein and small-molecule ligands was documented, and a complex simulation system was constructed. The construction of the simulation system was undertaken under constant temperature and pressure and with periodic boundary conditions. During the MD simulation, the LINCS algorithm was employed to constrain all hydrogen bonds with an integration step of 2 fs. The electrostatic interaction between the particle-mesh Ewald PME method and truncation at a value of 1.2 nm was also considered. The nonbonded interaction cutoff value was set to 10 Å and updated at 10 Å intervals. The V-rescale temperature coupling method was utilized for the control. The simulation temperature was set to 298 K, and the Berendsen method was employed to regulate the pressure to 1 bar. At 298 K, 100 ps of NVT and NPT equilibrium simulations were performed, and 100 ns of MD simulations were performed for the complex system, with conformations saved every 10 ps. Following the completion of the simulation, the simulated trajectory was analyzed using VMD and PyMOL, with the g_mmpbsa program utilized to perform MMPBSA between the protein and the small molecular ligand free energy analysis.

### 2.9 Cell Culture, IC_50_ Value, and Cell Proliferation Assays

#### 2.9.1 Cell Culture

A549 cells were cultured in F-12K medium supplemented with 10% fetal bovine serum (FBS), 2 mM l-glutamine, and 10 μ/mL penicillin–streptomycin under standard conditions (37 °C, 5% CO_2_).

#### 2.9.2 IC_50_ Value

A549 cells were cultivated in 96-well plates at an initial density of 5 × 10^3^ cells per well. The 4′-DMEP was obtained from Shanghai Dibo Biotechnology Co., Ltd. (Catalog Number: K756287) in powdered form. A stock solution was prepared by dissolving the compound in dimethyl sulfoxide (DMSO) at a concentration of 6 mM, which was subsequently diluted with culture medium to achieve the desired working concentrations. The 4′-DMEP concentrations used in the study ranged from 0 to 80 μM, with concentrations of 0, 5, 10, 20, 30, 40, 50, 60, 70, and 80 μM being used to treat the cells for 48 h. The cells constituting the control group were exposed to a concentration of 40 μM of the solvent DMSO. A quantity of MTT (1 mg/mL) was added to each well, and the resultant cell viability was measured on a microplate reader following a 4 h incubation period.

#### 2.9.3 Cell Proliferation Curve

A549 cells were seeded in 96-well plates at a density of 4 × 10^3^ cells per well. The concentration of 4′-DMEP ranged from 0 to 60 μM, with the cells undergoing treatment for 0, 24, 48, and 72 h. The control group comprised untreated cells and cells treated with 40 μM of DMSO. A quantity of MTT (1 mg/mL) was added to each well, and the resultant cell viability was measured on a microplate reader following a 4 h incubation period.

### 2.10 Flow Cytometry Assays

The cells were seeded in 6 cm cell culture dishes and cultured for 24 h. After the old medium was removed, the diluted drugs were added at concentrations of 20, 40, and 60 μM. The control group included untreated cells and cells treated with 0.5% DMSO. After 48 h of drug treatment, the conditioned medium was collected, and cells were detached using trypsin solution (EDTA-free) for 5 min at 37 °C. The cell suspension was centrifuged at 1000×*g* for 3 min, followed by two gentle washes with ice-cold 1× phosphate-buffered saline (PBS). Apoptosis analysis was performed strictly according to the protocol provided with the Annexin V-AbFluor 488/Propidium Iodide (PI) Apoptosis Detection Kit (Abbkine Scientific Co. Ltd., Catalog No. KTA0002). Cells were dual-stained with Annexin V-FITC and PI in binding buffer for 15 min under light-protected conditions. The samples were analyzed within 1 h poststaining to quantify apoptotic populations by LongCyte flow cytometry (Beijing Challen Biotechnology Co. Ltd.).

### 2.11 Western Blotting

Cells were seeded in 6 cm dishes and cultured for 24 h prior to treatment with 4′-DMEP at concentrations of 20, 40, and 60 μM. Control groups consisted of untreated cells and cells treated with vehicle and exposed to DMSO. Following 48 h of drug exposure, cells were lysed using RIPA lysis buffer supplemented with PMSF and protease inhibitor cocktail. The protein lysate was transferred to a PVDF membrane after SDS-PAGE gel electrophoresis. After blocking with 5% skimmed milk powder at room temperature for 1 h, the membrane was incubated with the primary antibody at 4 °C overnight. Antibodies against the following targets were obtained: TOP2A was from Beyotime (cat. no. AF0303); slc2A1 is derived from Servicebio (cat. no. GB113495); MIF was purchased from Wanleibio (cat. no. HA722674); and β-Actin (loading control) from Servicebio (cat.no.ZB15001-HRP). According to the standard immunoblotting procedure, protein signals were detected by enhanced chemiluminescence.

All experiments were performed in triplicate with three independent biological replicates. Statistical analyses were conducted using GraphPad Prism 9 software (version 9.0.0). Intergroup differences were evaluated through one-way analysis of variance (ANOVA) followed by Tukey’s post hoc multiple comparisons test. An unpaired Student’s *t-*test was applied for comparisons between independent experimental groups. A *p*-value <0.05 was considered statistically significant, with exact *p*-values reported for all quantitative analyzes.

## 3 Results

### 3.1 Target Screening and Functional Analysis

The targets of 4′-DMEP were searched in the targetnet, superpred, and BATMAN databases; 63, 98, and 19 targets were obtained, respectively. After the processes of deduplication and removal of irrelevant entries, a total of 171 targets were retained; 11439 LUAD-related genes were identified from the Genecards database. The intersection of the two gene sets was found to result in the identification of 131 potential genes for 4′-DMEP against LUAD (Figure 1A). Protein–protein interaction (PPI) networks were constructed and visualized using Cytoscape version 3.8.0. Initial analysis of 131 overlapping targets in the GEPIA database identified 38 genes with statistically significant differential expression (Figure 1B). Subsequent Kaplan–Meier (KM) survival analysis with a log-rank *p*-value threshold of <0.05 (including hazard ratio, HR) narrowed these to 18 core genes for further investigation (Figure 1C).

**Figure 1:**
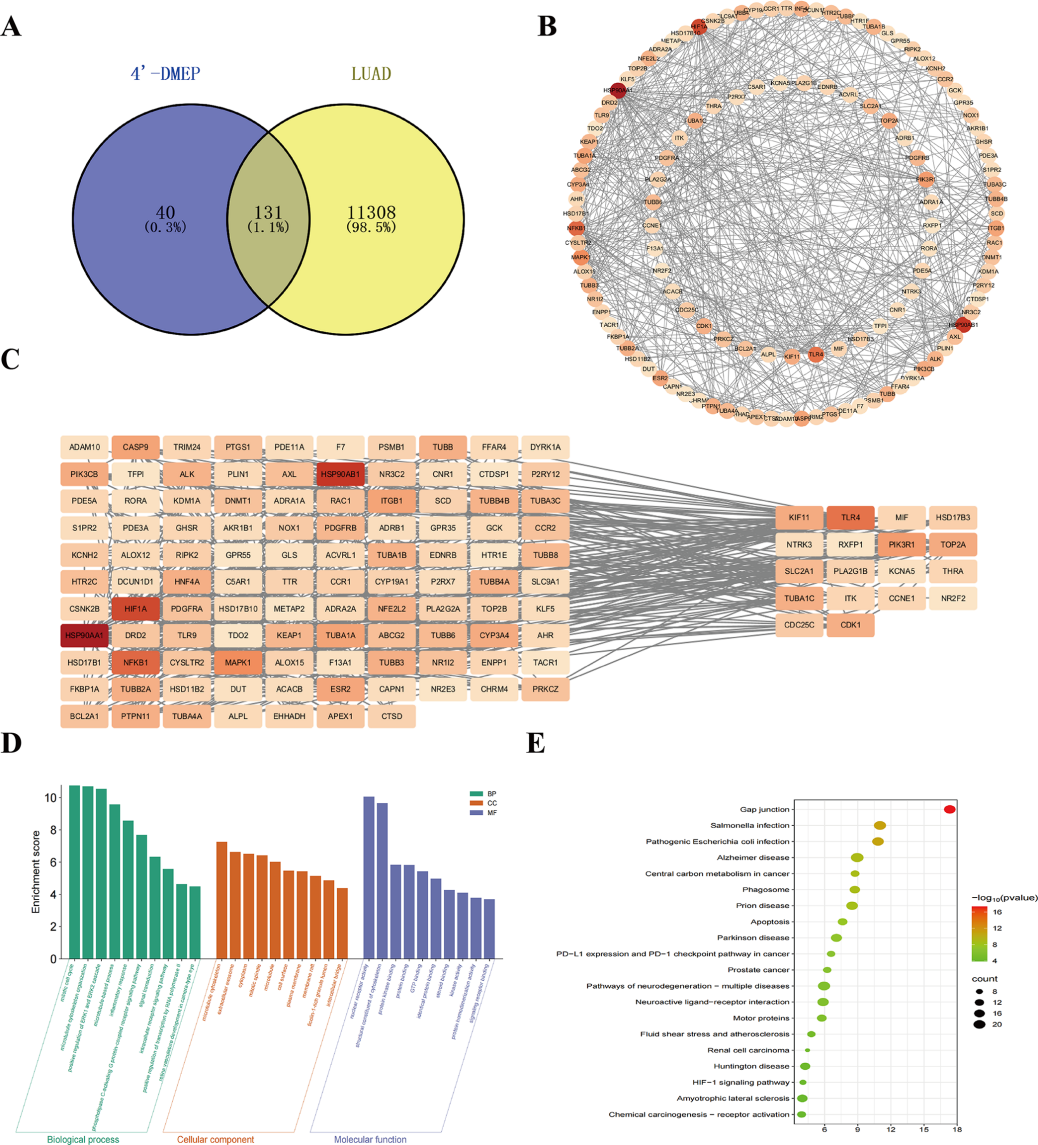
4′-DMEP and LUAD intersection target gene screening and GO/KEGG analysis. (A) Venn diagram illustrating overlapping targets between LUAD and 4′-DMEP. (B) Screening of significantly differentially expressed genes (DEGs) using the GEPIA database (|log2 fold change| > 1, adjusted *p* < 0.05). (C) Identification of core target genes through univariate Cox regression analysis (threshold: *p* < 0.05). (D, E) Functional enrichment analysis of overlapping targets; (D) Gene Ontology (GO) terms categorizing biological processes, molecular functions, and cellular components. (E) KEGG pathway enrichment (top 20 significant terms, *p* < 0.05).

Gene Ontology (GO) enrichment analysis of the 131 targets was performed using the DAVID database with biological processes (BP), cellular components (CC), and molecular functions (MF) systematically categorized (Figure 1D). The top ten significantly enriched terms (ranked by *p*-value) were visualized on the Weishengxin platform (Figure 1D). Key BP terms included mitotic cell cycle regulation, microtubule cytoskeleton organization, and the positive regulation of ERK1/ERK2 signaling mechanisms central to cell cycle control, cytoskeletal dynamics, and signal transduction. Enriched CC terms highlighted the microtubule cytoskeleton and extracellular exosome, while MF analysis revealed nuclear receptor activity, structural constituents of the cytoskeleton, and protein binding.

Kyoto Encyclopedia of Genes and Genomes (KEGG) pathway enrichment analysis on the Weishengxin platform (Figure 1E) identified these genes as being related to gap junctions, Salmonella infection, pathogenic *Escherichia coli* infection, and Alzheimer’s disease. The gap junction was the most statistically significant pathway (indicated in red) with substantial gene representation (denoted by the large bubble size). Gap junctions (GJs), critical structures for direct intercellular communication, are frequently dysregulated in malignancies. Impairments in gap junctional intercellular communication (GJIC) are strongly associated with tumor progression, driving uncontrolled proliferation and metastatic behavior. These findings collectively elucidate potential mechanisms underlying 4′-DMEP’s therapeutic effects in lung adenocarcinoma (LUAD).

### 3.2 Gene Expression and Clinical Correlation

Two transcriptomic data sets (GSE18842:45 normal vs 46 LUAD samples; GSE19188:65 normal vs 91 LUAD samples) were retrieved from the Gene Expression Omnibus (GEO) database using the search term “lung adenocarcinoma” with stringent data type and sample size filters. An independent validation cohort (GSE31210:20 normal vs 226 LUAD samples) was subsequently selected for external validation.

Differential expression analysis of the 18 previously identified core genes between LUAD and normal tissues in the discovery cohorts (GSE18842 and GSE19188) revealed 17 significantly differentially expressed core genes (SDECGs), including MIF, PLA2G1B, TOP2A, SLC2A1, TLR4, and NR2F2 (Figure 2A, B). These SDECGs, validated in human clinical samples, demonstrated an enhanced translational relevance and diagnostic potential. Chromosomal localization of the 4′-DMEP-associated core genes is illustrated in Figure 2C. Pairwise correlation analysis of SDECGs in LUAD tissues revealed robust intergene associations, with predominantly positive correlations observed across the cohort (Figure 2D, E). This coordinated expression pattern suggests functional synergy among these molecular targets in the LUAD pathogenesis.

**Figure 2:**
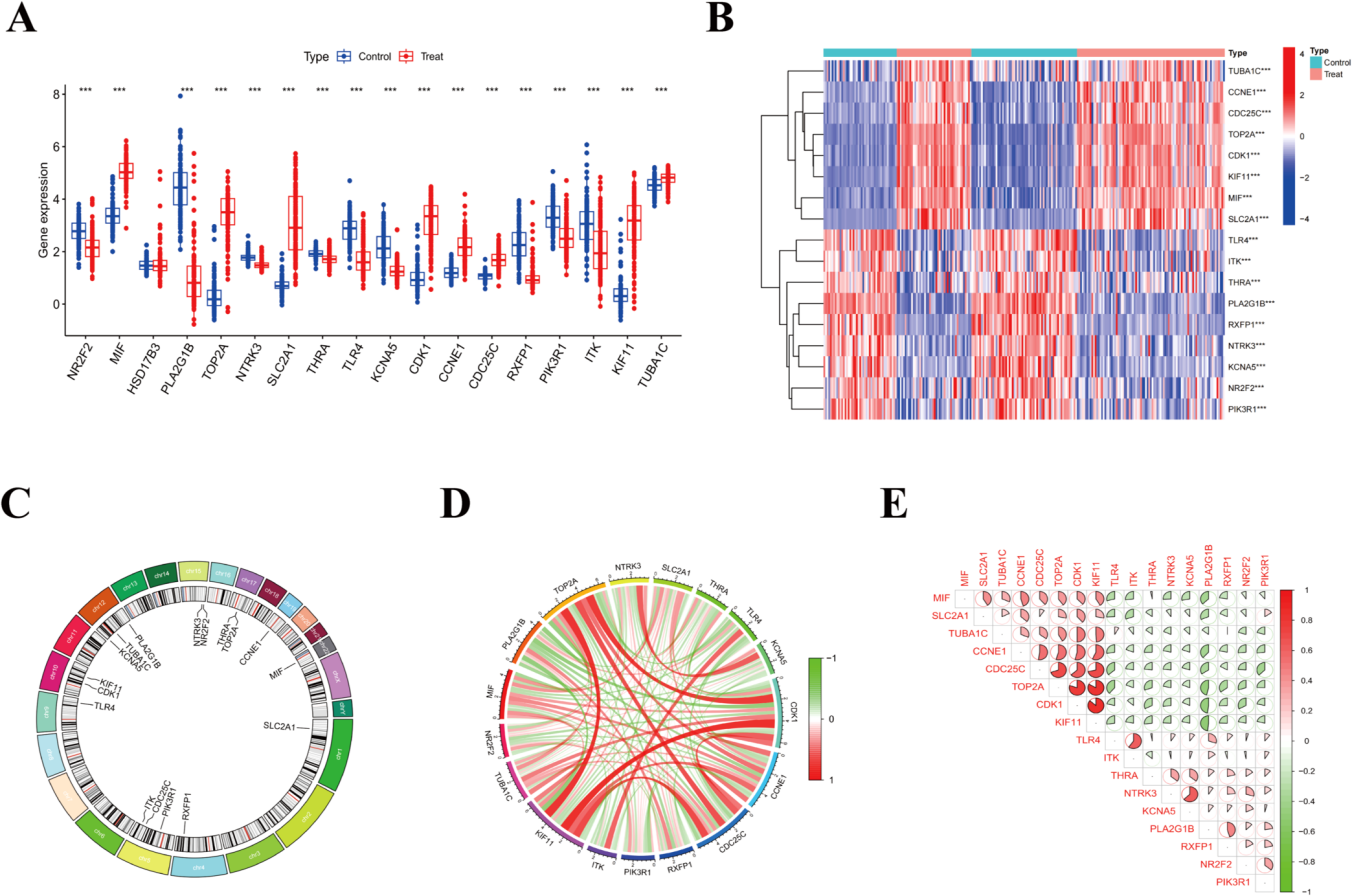
Expression and Interrelationships of Core Genes in LUAD. (A) Boxplot of differential expression analysis of core genes between normal and LUAD samples. (B) Heatmap of core gene expression in normal and LUAD samples. (C) Chromosomal locations of core genes. (D) Core genes correlation-associated network. (E) Correlation analysis between two of the core genes.

To investigate the alterations of the immune microenvironment in LUAD, we conducted a comprehensive analysis of immune cell infiltration to quantify subtype-specific immune cell populations.^26^ Compared with the normal control (*p* < 0.05, Figure 3A, B), the level of monocyte infiltration in LUAD tissues was significantly reduced. This finding was consistent with clinical evidence that decreased peripheral blood monocyte count is associated with advanced tumor progression and poor prognosis in LUAD patients. Mechanistically, monocyte depletion may promote tumorigenesis and metastatic dissemination by impairing the functional polarization of tumor-associated macrophages (TAMs), as shown in Figure 3B.^27^ The key genes were significantly correlated with immune cells in the tumor microenvironment, such as Toll-like receptor 4 (TLR4), which was positively correlated with eosinophils, M1 macrophages, monocytes, neutrophils, activated CD4 memory T cells, and γδT cells, which may regulate tumor immune surveillance/escape mechanisms by enhancing dendritic cell antigen presentation and T cell activation. SLC2A1 is positively correlated with naive CD4 T cells, which may affect immune cell metabolic reprogramming and antitumor function. PLA2G1B is positively correlated with monocytes, which may affect the immune response by regulating tumor microenvironment inflammation and cell signal transduction. MIF is significantly negatively correlated with γδT cells, which may promote tumor immune escape by inhibiting the activity of T cells and natural killer cells. ITK is positively correlated with M1 macrophages, CD8 T cells, activated CD4 memory T cells, and γδ T cells and negatively correlated with activated mast cells and NK cells, which may affect adaptive immunity by regulating T cell activation/differentiation. CDK1 is significantly negatively correlated with monocytes, which may affect the immune response by regulating the immune cell cycle and proliferation.

**Figure 3:**
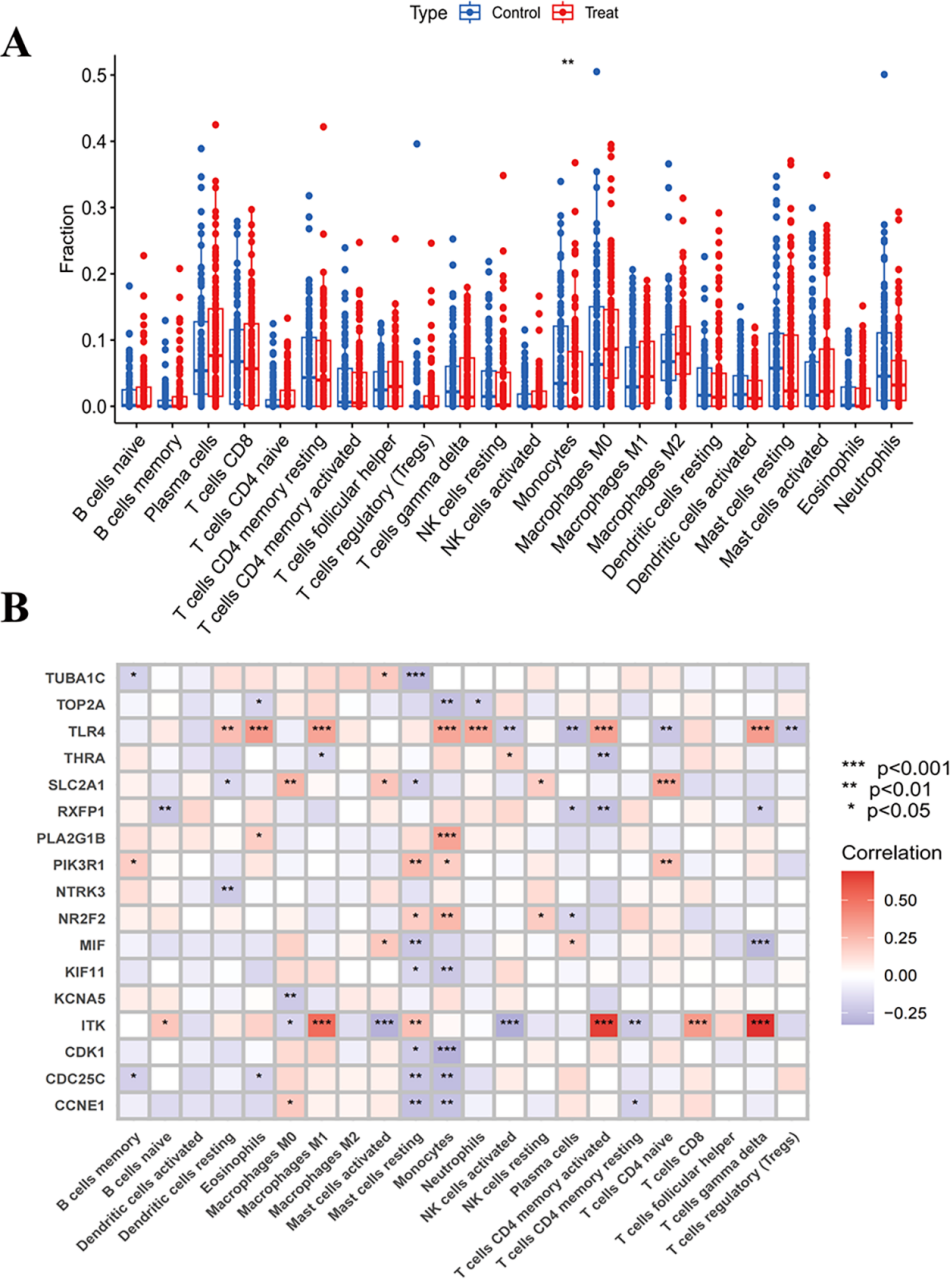
Association between core genes and LUAD immune infiltration. (A) Boxplot of immune cell scores between normal and LUAD samples. (B) Heatmap of correlation analysis between core genes and immune cells.

### 3.3 Machine Learning Model Construction

We evaluated four machine learning algorithms (support vector machine (SVM), random forest (RF), extreme gradient boosting (XGB), and generalized linear model (GLM)) using the 17 predefined SDECGs as predictive features. Model performance was systematically assessed on an independent testing set using multiple complementary evaluation approaches, including receiver operating characteristic (ROC) analysis, residual boxplots, reverse cumulative distribution plots, and confusion-matrix-based classification metrics.

Among the four models, the SVM demonstrated a superior and consistent predictive performance. It achieved the highest discriminative ability with an AUC of 0.993 (95% CI: 0.973–1.0), along with minimal residual dispersion and favorable reverse cumulative distribution characteristics (Figure 4A–C). Importantly, the robustness of the SVM model was further supported by confusion-matrix analysis, which yielded excellent classification performance, including high overall accuracy (98.6%), perfect sensitivity (100%), high specificity (96.97%), and a strong F1 score (0.988). RF exhibited comparable performance, whereas XGB and GLM showed relatively lower but still stable predictive accuracy. The concordance between ROC-based discrimination and classification-based metrics suggests that the observed high AUC values reflect a genuine predictive capability rather than overfitting. Based on this integrated evaluation, the SVM model was selected for subsequent analyzes.

**Figure 4:**
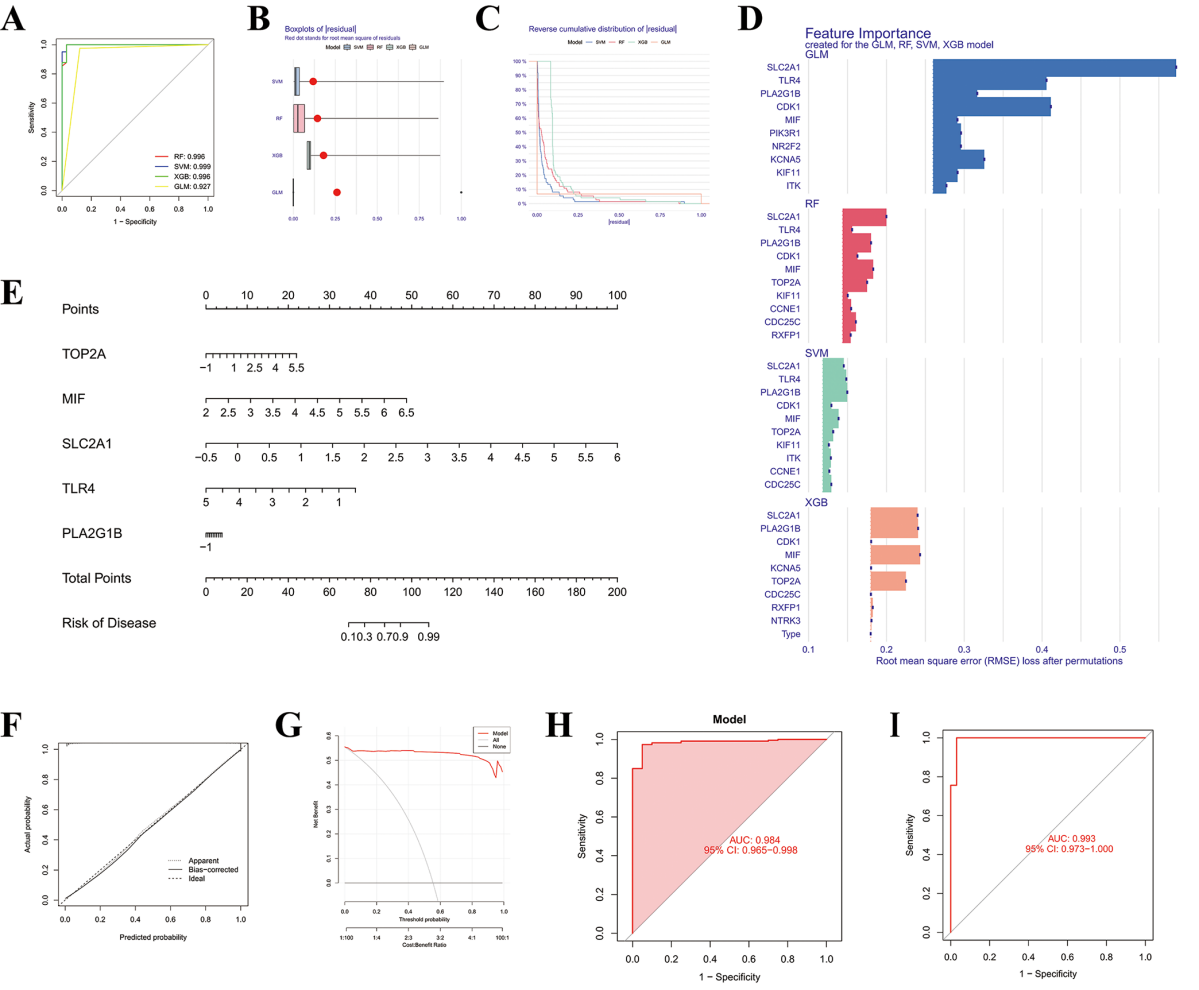
Machine learning screening of molecular targets for 4′-DMEP anti-LUAD. (A) ROC curves of four machine learning models. (B) Boxplots of residuals for the four machine learning models. (C) Reverse cumulative distribution of residuals for the four machine learning models. (D) Bar plots of feature importance for the four machine learning models. (E) Nomogram of the five feature genes. (F) Calibration curve of the feature gene model for 4′-DMEP-treated LUAD. (G) Decision curve of the feature gene profile for 4′-DMEP-treated LUAD. (H, I) ROC curves of the validation GEO data set.

Feature importance analysis within the SVM framework identified 10 key genes (SLC2A1, TLR4, PLA2G1B, CDK1, MIF, PIK3R1, NR2F2, KCNA5, KIF11, and ITK), with PLA2G1B ranking highest (Figure 4D). A multivariable clinical nomogram incorporating the top five genes (TOP2A, MIF, SLC2A1, TLR4, and PLA2G1B) was subsequently constructed to quantify individual gene contributions (Figure 4E). The nomogram-derived risk score, calculated as the weighted sum of expression levels of these signature genes, demonstrated a strong predictive performance. Model robustness was further supported by good agreement between predicted and observed outcomes in calibration analysis and favorable net benefit across a wide range of threshold probabilities in decision curve analysis (DCA), with an AUC of 0.993 (95% CI: 0.973–1.0).

External validation using the independent GSE31210 cohort (20 normal samples and 226 LUAD samples) further confirmed the generalizability of the model. The SVM-based model incorporating the top five feature genes achieved an AUC of 0.984 (95% CI: 0.965–1.0) in the external data set, demonstrating reproducible performance across data sets and supporting the robustness of the proposed predictive framework (Figure 4F–I).

### 3.4 Molecular Docking and Molecular Dynamics Simulation Validation

To verify whether 4′-DMEP has the greatest therapeutic effect on LUAD, we performed molecular docking studies on it. By molecular docking analysis of LUAD signature genes and 4′-DMEP, we found that the binding energy of the docking combination of these five core genes (TOP2A, MIF, SLC2A1, TLR4, and PLA2G1B) was lower than −5.0 kcal/mol (Table 1). There were hydrogen bonds between these five core targets and 4′-DMEP, indicating that a stable structure could be formed between all of the signature genes and 4′-DMEP (Figure 5). Therefore, we performed molecular dynamics simulations of these five core targets and concluded that the small molecule stably binds to the protein. The binding energy and affinity between 4′-DMEP and these target proteins were strong. The van der Waals interaction plays a major role, while the hydrophobic interaction and electrostatic interaction play a minor role.

**Figure 5:**
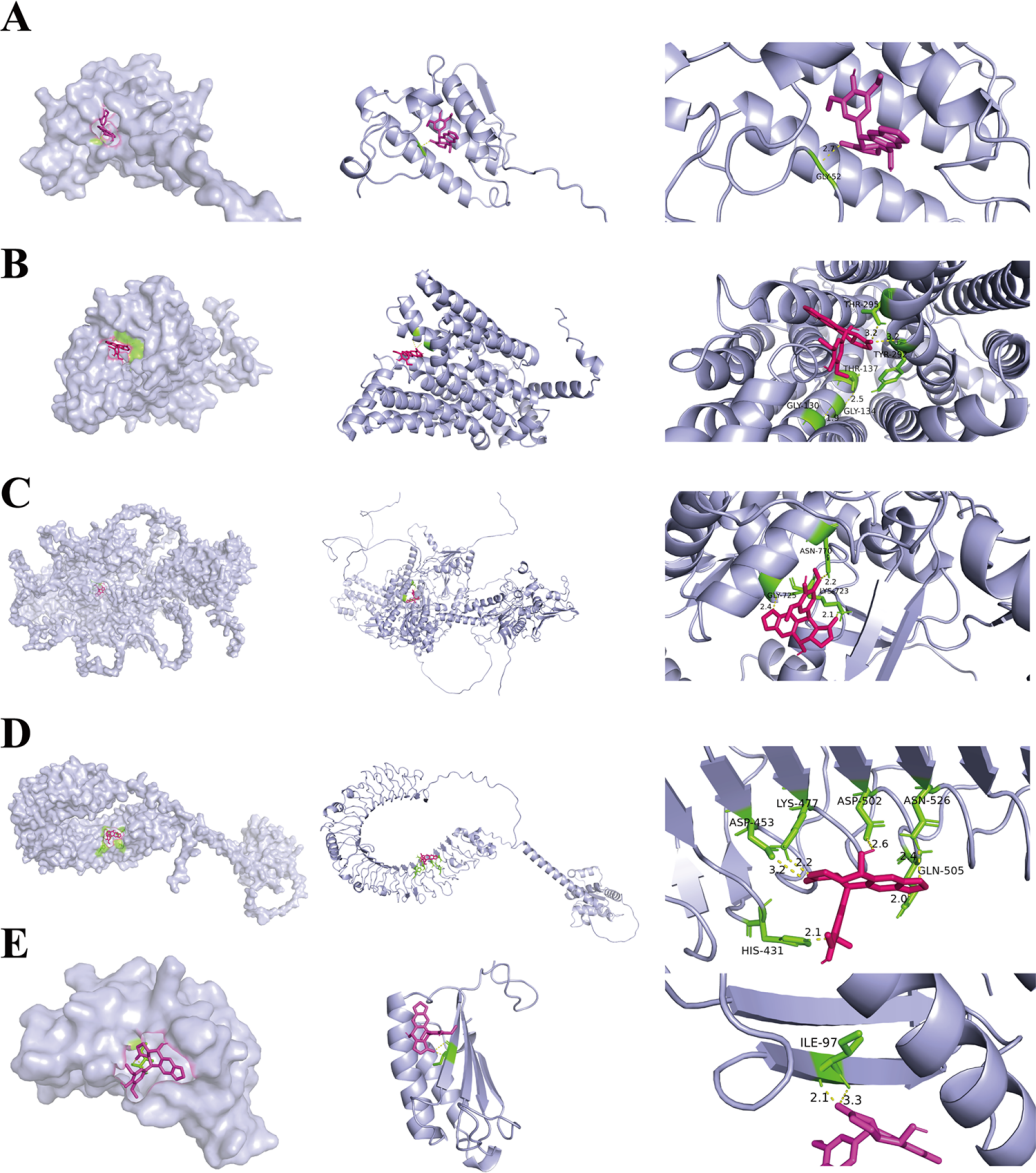
Molecular docking between 4′-DMEP and five target genes. AutoDock software was utilized to facilitate molecular docking of 4′-DMEP to the following proteins: PLA2G1B (A), SLC2A1 (B), TOP2A (C), TLR4 (D), and MIF (E).

**Table 1:** Binding Energy of Core Target between 4′-DMEP and LUAD^a^

gene	Uniprot ID	alphafold	binding energy (kcal/mol)	hydrogen bond
PLA2G1B	P04054	AF–P04054-F1	–8.2	GLY-52
SLC2A1	P11166	AF–P11166-F1	–7.7	THR-295, THR-137, TYR-292, GLY-130, GLY134
TOP2A	P11388	AF–P11388-F1	–7.6	ASN-770, GLY-725, LYS-723,
TLR4	O00206	AF–O00206-F1	–6.6	ASP-453, ASP-502, LYS-477, ASN-526, GLN-505, HIS-431
MIF	P14174	AF–P14174-F1	–6.1	ILE-97

a Note: The protein structures used for molecule docking were AlphaFold models sourced from UniProt.

To delve deeper into the stability of protein–ligand interactions, molecular dynamics (MD) simulations were conducted on five protein–ligand complexes: PLA2G1B-4′-DMEP, SLC2A1–4′-DMEP, TOP2A-4′-DMEP, TLR4–4′-DMEP, and MIF-4′-DMEP. The root-mean-square deviation (RMSD) was employed to evaluate whether the simulation systems attained a stable state, with RMSD values within 1 nm indicating the relative stability of protein–ligand interactions under physiological conditions. As illustrated in Figure 6A, the RMSD values of the five complexes rapidly stabilized at 0.28 ± 0.02 Å, 0.21 ± 0.02 Å, 0.30 ± 0.04 Å, 0.32 ± 0.14 Å, and 0.39 ± 0.08 Å, respectively.

**Figure 6:**
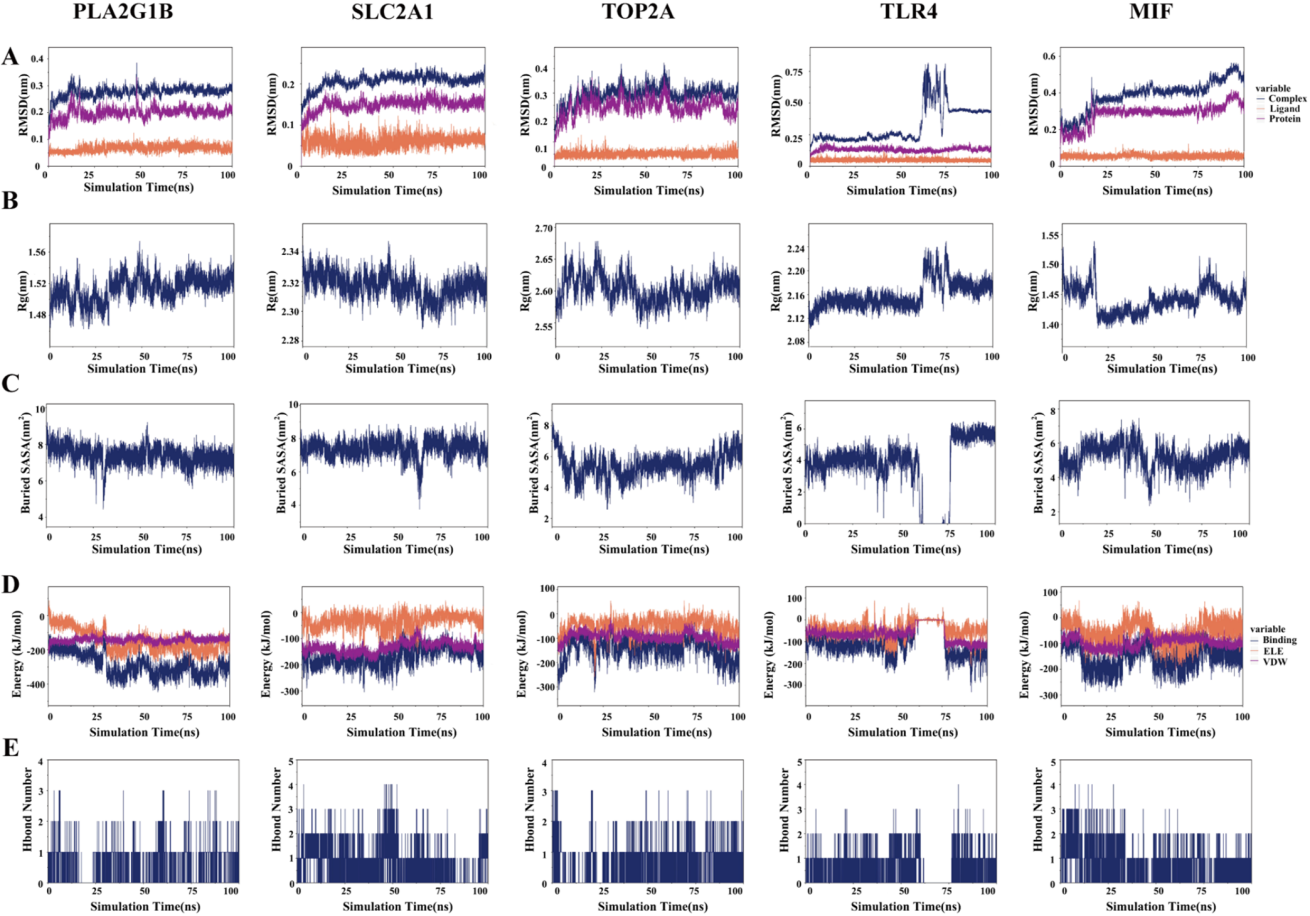
Results of molecular dynamics simulations. (A) Root-mean-square deviation (RMSD) evolution curves of the complex, protein, and small-molecule ligand over time. RMSD values reflect the overall conformational fluctuation of the system relative to the initial structure during the simulation. Low and stable values indicate that the system has reached an equilibrium state. (B) Radius of gyration (Rg) variation curve of the complex over time. Rg is used to measure the compactness of the complex structure. A stable Rg value indicates that the conformation of the complex remains compact without significant dissociation. (C) Variation curve of the buried solvent-accessible surface area (SASA) at the protein–ligand interface. The buried SASA value characterizes the strength of hydrophobic interactions at the binding interface. A larger and more stable value suggests the formation of a stable hydrophobic core at the binding interface. (D) Analysis of the binding energy components between the ligand and protein. VDW represents the contribution of van der Waals interactions, and ELE represents the contribution of electrostatic interactions. Together, they constitute the main components of the binding free energy calculated using molecular mechanics. (E) Dynamic statistics of the number of hydrogen bonds between the protein and ligand. The number and persistence of hydrogen bonds directly reflect the specificity and stability of the binding. The figure displays the dynamic characteristics of hydrogen bond formation during the simulation.

The radius of gyration (Rg) was analyzed to assess the compactness of receptor–ligand binding. As shown in Figure 6B, the Rg values of the complexes remained stable throughout the simulation, stabilizing at 1.51 ± 0.02, 2.32 ± 0.01, 2.60 ± 0.02, 2.16 ± 0.02, and 1.44 ± 0.02 nm, respectively. The solvent-accessible surface area (SASA), a key parameter reflecting protein folding and stability, also exhibited stability for the five complexes, with average values of 7.39 ± 0.50 nm^2^, 7.41 ± 0.52 nm^2^, 5.43 ± 0.78 nm^2^, 3.718 ± 1.806 nm^2^, and 5.220 ± 0.70 nm^2^, respectively (Figure 6C). The number of hydrogen bonds, an indicator of protein–ligand binding strength, was measured to determine the density and stability of the hydrogen bonds (Figure 6D).

Furthermore, the binding free energies (ΔGbind) of the four protein–ligand complexes were calculated using the MM/PBSA method, where lower Δ*G* bind values signify stronger receptor–ligand binding affinity.^30^ As depicted in Figure 6E, the ΔGbind rankings of the four complexes were TOP2A-4′-DMEP (−8.230 ± 0.299 kcal/mol) > TLR4–4′-DMEP (−9.000 ± 1.679 kcal/mol) > MIF-4′-DMEP (−11.069 ± 0.689 kcal/mol) > SLC2A1–4′-DMEP (−12.365 ± 1.459 kcal/mol) > PLA2G1B-4′-DMEP (−13.821 ± 1.440 kcal/mol), which aligns with the molecular docking results.

### 3.5 In Vitro Experimental Validation

The IC_50_ value of 4′-DMEP in A549 cells after 48 h treatment was determined to be 53.96 ± 1.833 μM (Figure 7A). The cell proliferation curve in Figure 7B showed that following treatment with 4′-DMEP at the indicated time points (24, 48, and 72 h), cell viability significantly decreased in a dose-dependent manner. Moreover, as shown in Figure 7B, the experimental results of the DMSO group were comparable to those of the control group, indicating that DMSO had no significant effect on the experimental outcomes. Cell cycle analysis of A549 cells after 4′-DMEP treatment for 48 h demonstrated a significant increase in the proportion of cells in the G1 phase and a corresponding decrease in the G2 phase (Figure 7C). Notably, the prolongation of the G1 phase induced by 4′-DMEP exhibited a clear dose-dependent trend (Figure 7E). Annexin V-AbFluor 488/PI apoptosis assays revealed that treatment with 20 μM, 40 μM, and 60 μM of 4′-DMEP induced apoptosis rates of (13.53 ± 1.86)%, (15.84 ± 1.32)%, and (18.77 ± 1.52)% in A549 cells, respectively (Figure 7D). These values were significantly higher than those in normal control cells (3.22 ± 0.30)% and the DMSO group (3.54 ± 0.42)% (Figure 7F). The 20 μM concentration of the drug exerted a significant effect on early apoptosis in A549 cells, with a Mean Difference of −3.453333 (95%CI −5.238343 to −1.668324; *p* = 0.001536). Although the treatment with 4′-DMEP increased total apoptosis, a significant difference was observed in the distribution between the early and late stages of apoptosis (Figure 7D). This indicated that 4′-DMEP can induce cell apoptosis, and its regulatory effect shows significant differences in both the overall apoptotic level and specific stages of early and late apoptosis. The results suggested that 4′-DMEP may affect the apoptosis pathway at multiple nodes, potentially by activating upstream pro-apoptotic signals while promoting the transition of cells from early to late apoptosis.^29,30^

**Figure 7:**
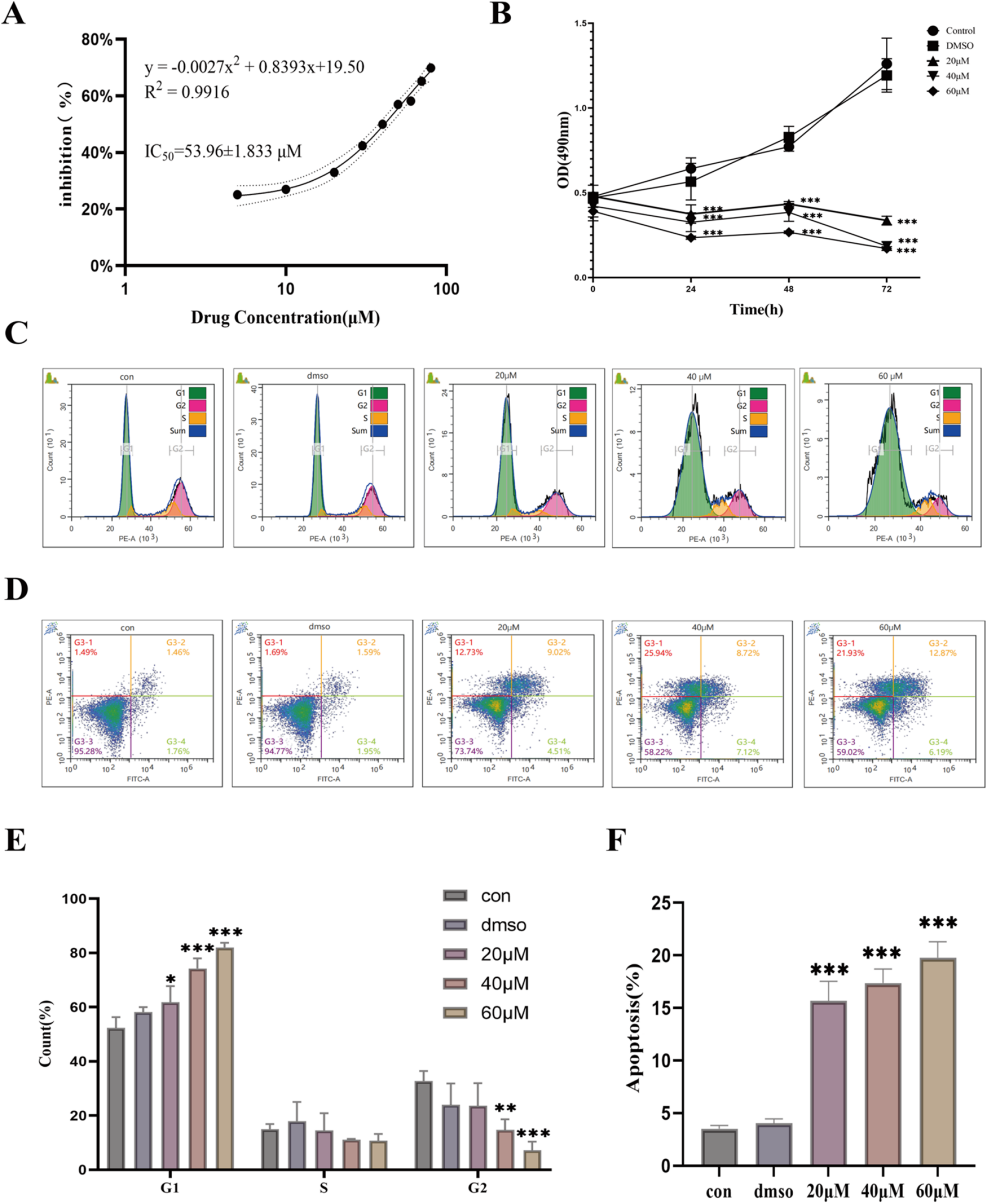
4′-DMEP inhibits the proliferation of A549 cells. (A) The IC_50_ value of 4′-DMEP on A549 cells. (B) The proliferation curve of different concentrations of 4′-DMEP. (C) 4′-DMEP blocked the cell cycle. (D) 4′-DMEP induced cell apoptosis. Histogram statistics of cell cycle ratios (E) and apoptosis rates (F). The data are presented as the mean ± SD of three independent experiments (*n* = 3), with *p ≤ 0.05, **p ≤ 0.01, and ***p ≤ 0.001 indicating statistical significance. Con, no-treated group; DMSO, DMSO-treated group; 20–60 μM, 4′-DMEP-treated group.

To validate the molecular docking and dynamics simulation results, we performed Western blot analysis on the top three candidate proteins exhibiting the highest binding affinities for 4′-DMEP: SLC2A1, TOP2A, and MIF. Dose–response experiments in A549 cells demonstrated concentration-dependent regulation patterns: both MIF and SLC2A1 protein expression showed progressive downregulation, while TOP2A expression was significantly upregulated compared with untreated controls and vehicle (DMSO)-treated groups. This dose-dependent modulation across all three targets strongly suggests that 4′-DMEP directly influences the expression levels of these proteins through specific pharmacological interactions (Figure 8).

**Figure 8:**
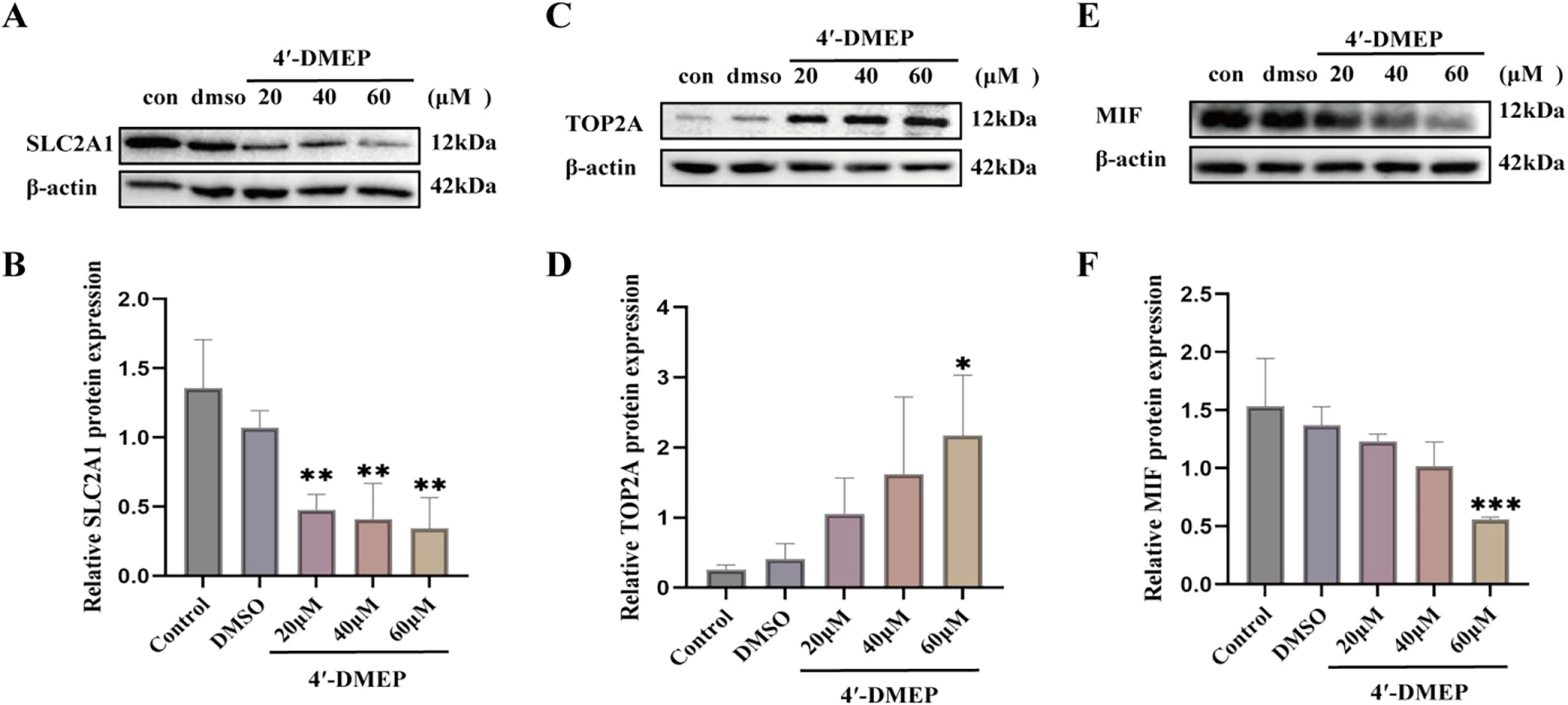
4′-DMEP affected the expression of target proteins SLC2A1, TOP2A, and MIF. Western blotting detected the expression of SLC2A1 (A), TOP2A (C), and MIF (E) protein in different concentrations of 4′-DMEP-treated A549 cells. The data (B, D, F) are presented as the mean ± SD of three independent experiments (*n* = 3), with *p ≤ 0.05, **p ≤ 0.01, and ***p ≤ 0.001 indicating statistical significance. Con, no-treated group; DMSO, DMSO-treated group; 20–60 μM, 4′-DMEP-treated group.

## 4 Discussion

In this study, we successfully predicted the potential key targets (SLC2A1, TOP2A, MIF, TLR4, PLA2G1B) of 4′-DMEP in LUAD by integrating network pharmacology, machine learning, and molecular simulation. In vitro experiments confirmed that 4′-DMEP could significantly inhibit the proliferation of A549 cells by inducing cell cycle arrest and apoptosis, and the expression changes of SLC2A1, TOP2A, and MIF were verified by Western blotting. This evidence suggested that 4′-DMEP exerted its anti-LUAD effects through a multitarget and multipathway, and its core mechanism was related to interference with cell cycle, apoptosis, and energy metabolism. The integrated findings from machine learning, molecular docking, and molecular dynamics simulations collectively demonstrate a coherent mechanism underlying 4′-DMEP’s anti-LUAD efficacy. Molecular docking revealed high-affinity binding between 4′-DMEP and key targets (SLC2A1, TOP2A, MIF, TLR4, PLA2G1B). Molecular dynamics simulations further confirmed the stability of these complexes, showing low RMSD fluctuations, persistent hydrogen bonding, and favorable free energy values (Δ*G* < – 30 kcal/mol), indicating sustained target engagement.

SLC2A1 is a key carrier protein that mediates glucose transmembrane transport and plays a central role in tumor metabolic reprogramming, especially glucose metabolic reprogramming.^31^ Its high expression in NSCLC enhances glucose uptake capacity, efficiently supporting the Warburg effect in tumor cells and promoting lactate accumulation and tumor microenvironment acidification. Identification of SLC2A1 is a predictive biomarker for survival and response to immunotherapy in lung squamous cell carcinoma. Computers in Biology and Medicine, 108183.^32^ This ultimately drives tumor cell proliferation, and knockout of the SLC2A1 protein reduces glucose uptake and glycolysis flux. This metabolic stress leads to cell arrest in the G1/S phase, increases intracellular ROS levels, and activates cell apoptosis.^33^ As a key enzyme that regulates DNA replication and repair, TOP2A exhibits significantly elevated expression in NSCLC.^34^ Abnormal expression of TOP2A is closely associated with the malignant biological behaviors of tumors, such as invasion and metastasis, and epithelial-mesenchymal transition (EMT), and it also directly affects the prognosis of patients by driving tumor progression. Clinical studies have confirmed that the expression level of TOP2A is positively correlated with the TNM stage and lymph node metastasis of NSCLC.^35^ MIF is a multifunctional cytokine that was initially characterized for its ability to inhibit macrophage migration. Subsequent studies have revealed its critical role in immune regulation, inflammatory responses, and tumor development and progression.^36^ In the context of lung cancer, MIF exerts a multifaceted role in the initiation, progression, metastasis, and treatment resistance of the disease. This involvement occurs through several mechanisms, including the regulation of the biological behavior of tumor cells, the remodeling of the tumor microenvironment, and the interference with antitumor immunity.^37^ Although TLR4 and PLA2G1B have not been verified, their known functions in inflammation and cancer are consistent with the results of the KEGG enrichment analysis. These findings suggested that 4′-DMEP exerted its anti-LUAD effects through a polypharmacological mechanism, providing a translatable paradigm for the development of natural-product-based therapies for LUAD.

In vitro experiments, 4′-DMEP, exhibited dose-dependent cytotoxicity in A549 cells (IC_50_ = 53.96 ± 1.833 μM). Some epipodophyllotoxin derivatives showed better cytotoxicity in A549, such as etoposide (IC_50_ = 9.32 ± 0.55 μM), [3′,4′:6,7]­naphtho­[2,3-*d*]­[1,3]­dioxol-5-yl4-(4-((3-bromophenyl)­sulfonamido)­phenyl)­butanoate (IC_50_ = 0.34 ± 0.13 μM), (5R,5aR,8aS,9R)-9-(4-cinnamoylpiperazin-1-yl)-5-(3,4,5-trimethoxyphenyl)-5,8,8a,9-tetrahydrofuro­[3′,4′:6,7]­naphtho [2,3-*d*]­[1,3]­dioxol-6­(5aH)-one (IC_50_ = 0.34 ± 0.13).^38–40^

During the interphase of the cell cycle, the G1 phase (macromolecular synthesis and growth), S phase (DNA replication), and G2 phase (predivision checkpoint) collectively ensure the efficacy and accuracy of cellular proliferation through the ordered execution of three critical functions: cellular growth, genetic material duplication, and quality surveillance mechanisms. The observed G1 phase cell cycle arrest (82.02% vs 52.28% in control cells) is consistent with prior reports on microtubule-disrupting agents in non-small cell lung cancer (NSCLC), such as vinorelbine, which induce checkpoint activation via TOP2A-mediated DNA damage.^41^ The dual induction of early (Annexin V+/PI-) and late (Annexin V+/PI+) apoptotic populations by 4′-DMEP suggests the sequential engagement of distinct signaling pathways.

Notably, unlike the downregulated expression of SLC2A1 and MIF, the expression of TOP2A was significantly upregulated after 4′-DMEP treatment. This seemingly contradictory phenomenon may be related to the feedback regulation mechanism of cells under DNA damage stress. As a topoisomerase II inhibitor, 4′-DMEP forms a stable ’cleavage complex’ with TOP2A, resulting in DNA double-strand breaks.^35^ This severe DNA damage activates DNA damage response pathways such as ATM/ATR-Chk1/Chk2 in cells.^42^ As a compensation, cells may attempt to upregulate the expression of TOP2A to compensate for TOP2A molecules that are functionally inactivated due to drug “capture”, in an attempt to restore normal DNA metabolism and maintain survival. However, this compensatory upregulation is likely to be futile because the newly synthesized TOP2A will continue to be inhibited by 4′-DMEP, eventually leading to the accumulation of DNA damage exceeding the threshold, thereby triggering the cell cycle arrest and apoptosis we observed.

The accuracy and reliability of core target screening were systematically improved by integrating network pharmacology, machine learning, molecular docking, molecular dynamics simulation, and in vitro experiments, which provided a feasible technical path for the systematic analysis of complex disease mechanisms. By targeting TOP2A (disrupting DNA topology), SLC2A1 (inhibiting glucose metabolism), and MIF (regulating the immune-inflammatory microenvironment), 4′-DEMP induces cell cycle arrest and apoptosis, thus constructing a multitarget and multipathway synergistic regulatory network, which deepens the understanding of the multidimensional antitumor mechanism of natural lignans. In addition, the identified target genes are expected to be potential biomarkers for prognosis evaluation or efficacy prediction of lung adenocarcinoma. This not only provides the basis of patient stratification and the target of structural optimization for the clinical transformation of 4′-DMEP but also develops a new candidate direction for the precise treatment strategy of lung adenocarcinoma. However, there are still some limitations in this study, such as the use of only one cell line for experiments and the lack of in vivo data support. Subsequent studies should further evaluate the efficacy of 4′-DMEP in a variety of lung adenocarcinoma cell lines and systematically evaluate its efficacy, dose response, and preliminary safety in vivo using a mouse xenograft model, so as to provide key data for the transformation of 4′-DMEP from in vitro research to clinical research. In the anticancer mechanism and signal pathway analysis of 4′-DMEP, the integrated application of multiomics methods will become the core technical support. By fusing the expression characteristics of multidimensional molecules such as genes, proteins, and metabolism, it is expected to further reveal the molecular mechanism of regulating the biological behavior of tumor cells. In summary, 4′-DMEP, as a candidate molecule for the treatment of lung adenocarcinoma derived from natural products, shows the potential to simultaneously interfere with proliferation and apoptosis pathways through multitarget pharmacological properties. In the future, the combination of patient-derived organoid models and multiomics analysis will be the key to verifying their clinical potential and identifying predictive markers of treatment response.

## 5 Conclusion

In this study, the potential targets of 4′-DMEP against lung adenocarcinoma were screened based on network pharmacology and further screened by PPI network analysis and machine learning methods. Finally, TOP2A, MIF, SLC2A1, TLR4, and PLA2G1B were identified as the top five key targets, which were verified by molecular docking and molecular dynamics simulation analysis. In addition, in vitro experiments using A549 cells treated with different concentrations of 4′-DMEP provided experimental support for the above findings. Overall, our results highlight the potential of 4′-DMEP as a candidate molecule for the treatment or prevention of lung adenocarcinoma, laying an important foundation for subsequent research. It is worth noting that further pharmacological and clinical studies are essential to verifying the above conclusions.

## Supplementary Material



## Data Availability

The data underlying this study are available within the manuscript and its accompanying Supporting Information files.

## Notes

The URLs of Web sites and Public Databases; the version of software and Analysis Tools; the Original picture of protein immunoblotting experiment of MIF, TOP2A, and SLC2A1 (PDF)
