# Supplementary material for: Deciphering the Anti-LUAD Mechanism of 4′-Demethyl-epipodophyllotoxin (4′-DMEP) via Machine Learning-Driven Target Identification and In Vitro Validation
Source: ACS Omega. 2026 Apr 3;11(15):22983–96. doi: 10.1021/acsomega.5c12467 (PMC13103775; doi:10.1021/acsomega.5c12467)
Supplement: Supplementary file 1 [file ao5c12467_si_001.pdf]

Supplementary Material

Deciphering the Anti-LUAD Mechanism of 4'-Demethyl-epipodophyllotoxin (4'-DMEP) via Machine Learning-Driven Target Identification and in vitro validation

Jinghui Yao[a], Chenhao Wang[a], Zhichao Wang[a], Shi Xiang[a], Wu Sun[a], Hui Chen[a,b], Chao Yang[a,b*]

[a] Oncology Research Center, Jiangxi Provincial Key Laboratory of Traditional Chinese Medicine Diagnosis and Rehabilitation of Malignant Tumors, Jiangxi University of Traditional Chinese Medicine, Nanchang 330004, P. R. China

[b] Jiangxi Engineering Research Center for Translational Cancer Technology, Jiangxi University of Traditional Chinese Medicine Nanchang 330004, P. R. China

[*] Email: yangchao@jxutcm.edu.cn (C. Yang).

Table S1. URLs of Websites and Public Databases Used in the Study

Website name	Website URL
PubChem	https://pubchem.ncbi.nlm.nih.gov/
UniProt Database	https://www.uniprot.org
GeneCards Database	https://www.genecards.org/
DAVID Database	https://david.ncifcrf.gov/
Microbiome Online Analysis Platform	http://www.bioinformatics.com.cn
GEPIA Database	http://gepia.cancer-pku.cn/
GEO Database	https://www.ncbi.nlm.nih.gov/geo
PDB Database	http://www.rcsb.org/
TargetNet	http://targetnet. scbdd.com/
SuperPred	https://prediction.charite.de/
BATMAN	http://bionet.ncpsb.org/batman-tcm/
OMIM	https://www.omim.org/
TTD	http://db.idrblab.net/ttd/
Venny 2.1.0	https://bioinfogp.cnb.csic.es/tools/venny/index.html
RCSB PDB	https://www.rcsb.org/pages/about-us/index

Table S2. The version Software and Analysis Tools

Tool Name	Version
Cytoscape	3.8.0
R Language	4.4.1
GSVA (R package)	2.0.7
GSEABase (R package)	1.68.0
limma (R package)	3.62.2
pheatmap (R package)	1.0.12
ggpubr (R package)	0.6.0
caret (R package)	7.0.1
randomForest (R package)	4.7.1.2
kernlab (R package)	0.9.33
xgboost (R package)	1.7.11.1
pROC (R package)	1.18.5
DALEX (R package)	2.4.3
AutoDock Vina	1.2.3
PyMOL	2.5.7
GROMACS	2022
VMD	1.9.3
ggplot2	3.5.2
corrplot	0.95
circlize	0.4.16

The following is supplementary material for Figure 8，the gel images provided here are all raw.

Figure S1.These are supplementary materials for Figure 8A (MIF), showing data from three independent replicate experiments used for statistical analysis. This set of images represents the raw data presented in this article， for Figure 8A (MIF).

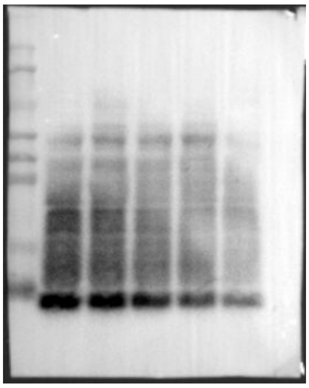

MIF

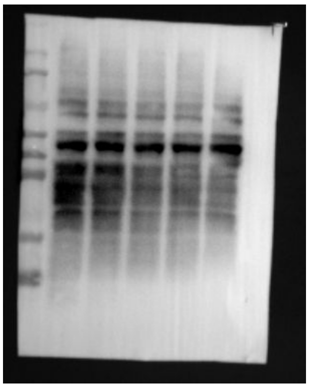

β-actin

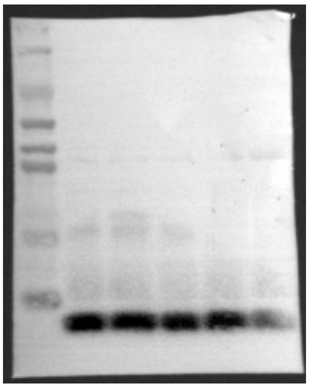

MIF

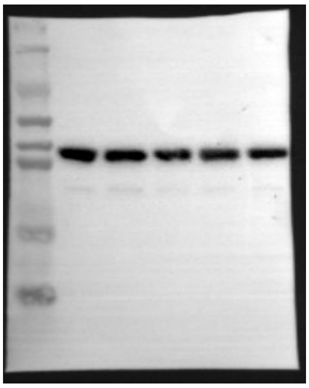

β-actin

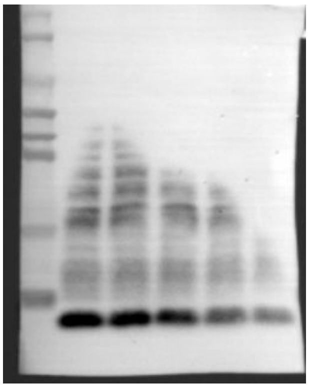

MIF

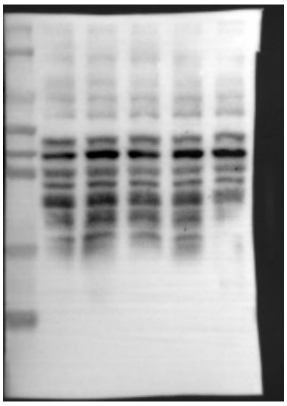

β-actin

Figure S2.These are supplementary materials for Figure 8C (TOP2A), showing data from three independent replicate experiments used for statistical analysis. This set of images represents the raw data presented in this article,for Figure 8C (TOP2A).

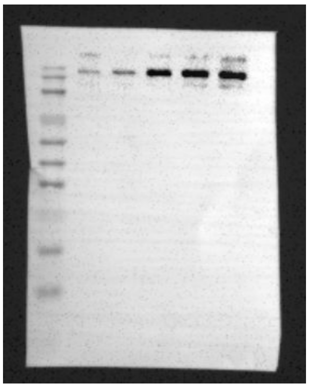

TOP2A

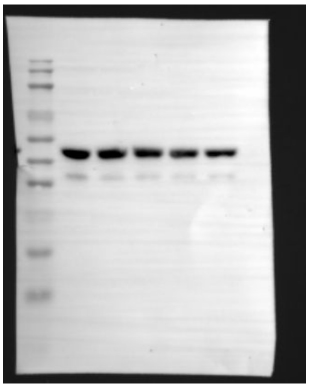

β-actin

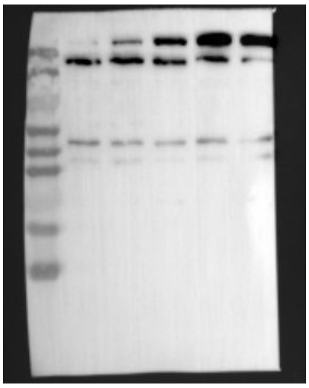

TOP2A

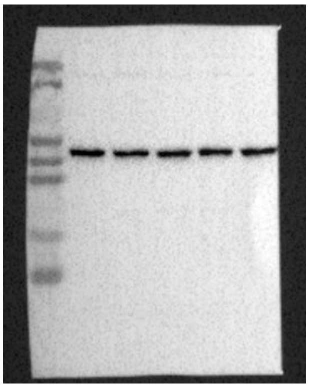

β-actin

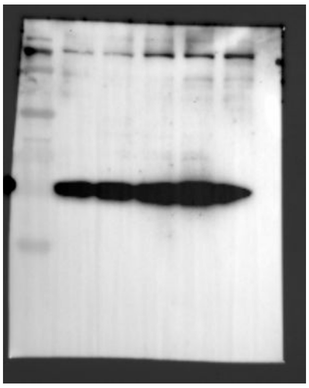

TOP2A

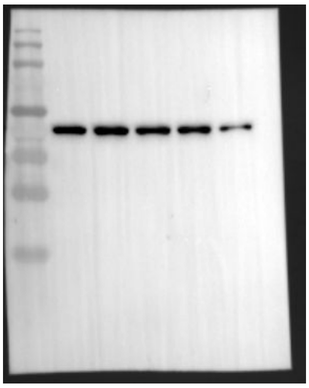

β-actin

Figure S3.These are supplementary materials for Figure 8E (SLC2A1), showing data from three independent replicate experiments used for statistical analysis. This set of images represents the raw data presented in this article,for Figure 8E (SLC2A1).

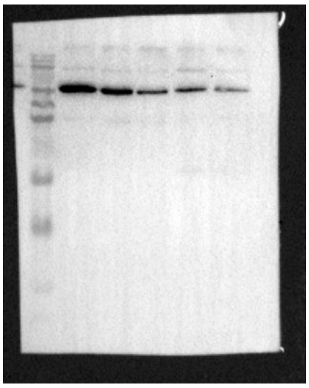

SLC2A1

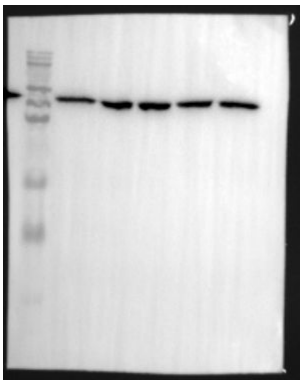

β-actin

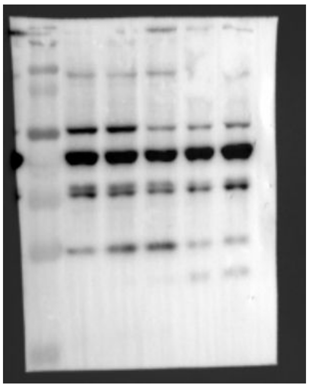

SLC2A1

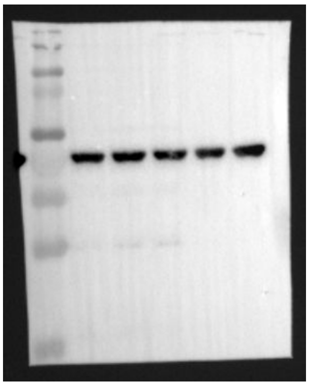

β-actin

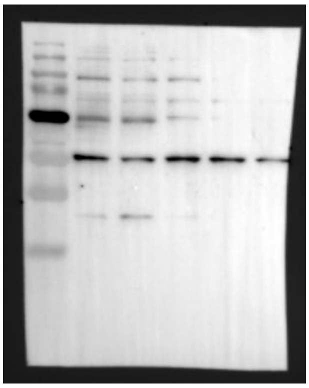

SLC2A1

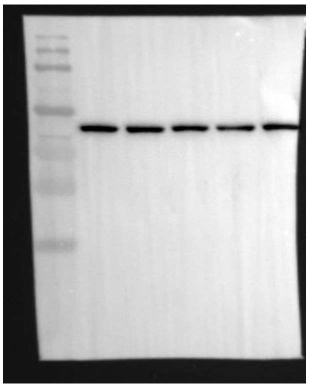

β-actin
